# Maximization of Non-Opioid Multimodal Analgesia in Ambulatory Surgery Centers

**DOI:** 10.7759/cureus.10407

**Published:** 2020-09-12

**Authors:** Karina Charipova, Kyle L Gress, Ivan Urits, Omar Viswanath, Alan D Kaye

**Affiliations:** 1 Medicine, MedStar Georgetown University Hospital, Georgetown University School of Medicine, Washington D.C., USA; 2 Anesthesiology, Beth Israel Deaconess Medical Center, Harvard Medical School, Boston, USA; 3 Anesthesiology, University of Arizona College of Medicine, Phoenix, USA; 4 Anesthesiology, Louisiana State University Health Sciences Center, Shreveport, USA

**Keywords:** pain management, ambulatory surgical procedures, outpatients, enhanced recovery after surgery, patient satisfaction, anesthestics

## Abstract

Ambulatory surgery centers aid the healthcare system by not only providing a cost-effective option for delivery of care but also by helping to reduce overwhelming case volumes at inpatient facilities. While outpatient protocols have been designed for an increasing number of surgical procedures, the inpatient to outpatient transition of surgery remains limited by both procedure type and patient comorbidities. This limitation stems in part from the heavy emphasis on accelerated discharge following outpatient procedures, given that prolonged recovery time is associated with delayed turnover and increased nursing care demands. Since its inception, enhanced recovery after surgery (ERAS) has aimed to primarily reduce the disruption of physiologic homeostasis that occurs secondary to surgery. More recently, the aim of ERAS has evolved to help transition inpatient procedures to outpatient settings and may even be useful in more emergent cases. It should be noted, however, that outpatient surgery even in combination with ERAS is not the best option for all patients, and the use of ERAS protocols should be complemented with predictive assessments of patient risk. Beyond augmenting the efficiency of outpatient surgery, ERAS protocols, when used in eligible patients and especially when combined with regional anesthetic techniques, are effective in delivering opioid-sparing pain management while increasing overall outcomes and patient satisfaction rates.

## Introduction and background

Ambulatory surgery centers allow procedures that are traditionally performed on an inpatient basis to be conducted as same-day outpatient surgeries [1]. In doing so, ambulatory surgery centers represent a cost-effective element in modern surgical care that has the potential to improve both outcomes and patient satisfaction rates [1-3]. One of the caveats of outpatient surgery, however, is prolonged recovery time associated with the use of anesthetic techniques beyond local analgesia. Prolonged recovery time creates issues with bed occupancy and unmanageable demands of nursing care [1]. Enhanced recovery after surgery (ERAS) was first introduced in 1997 as an evidence-based, multidisciplinary perioperative approach to caring for the surgical patient [4-8]. The main goal of ERAS is to accelerate recovery without increasing morbidity [7,9]. The Enhanced Recovery After Surgery Society is an international nonprofit organization that makes recommendations for delivering ERAS programs to patients undergoing various types of surgery, ranging from gynecologic procedures to pancreaticoduodenectomy [5].

ERAS pathways have traditionally been implemented in elective inpatient surgeries, but have become a topic of interest for ambulatory surgery centers given the expansion of their scope to more complex procedures [3]. In fact, it is believed that use of ERAS protocols can help transition inpatient procedures to outpatient settings since less invasive procedures and alternative approaches to anesthesia are conducive to ambulatory centers [7,10]. ERAS aims to reduce surgical stress on the body and to minimize disruption of anabolic homeostasis [3,4,6,8]. Implementation of ERAS guidelines has allowed institutions to decrease length of stay, rates of complications, and medical costs [4,5,11]. Of note, the integration of short-interval postoperative follow-up appointments and use of multimodal pain therapy into ERAS can help patients limit postoperative narcotic use [2]. These postoperative recovery protocols have been demonstrated to have good outcomes in elective inpatient surgery, and their use has been more recently successfully initiated at ambulatory surgery centers for procedures such as mastectomy with or without reconstruction, robotic prostatectomy, and thyroidectomy [3,4]. The implementation of ERAS into emergency settings continues to be a work in progress [4].

## Review

Key components of ERAS

The reaction of the body to surgery consists of a physiologic chain reaction that causes a systematic release of cytokines, free radicals, hormones, and inflammatory mediators that make up a stress response [3,7]. During this process, patients are initially at risk of becoming weak, immobile, and catabolic and can subsequently experience impaired healing, immunosuppression, postsurgical complications [3,7,8]. ERAS methods differ from conventional approaches to perioperative care mainly in their emphasis on accelerating recovery by decreasing the physical and psychological responses of the body to surgical stress [3,5,6]. Specific goals of ERAS include minimizing primary surgical injury and blood loss through using minimally invasive techniques, implementing pharmacotherapy like tranexamic acid, individualizing fluid therapy to decrease risk of gut ileus, optimizing pain control with multimodal analgesia, and encouraging early postoperative mobilization to decrease risk of atelectasis, pneumonia, and deep vein thrombosis [3,7].

ERAS guidelines make adjustments to each of the three phases of surgery [3,5,8]. Preoperatively, the focus is on properly preparing the patient for surgery without unnecessary disruptions to homeostasis. This phase includes patient education and both fluid and carbohydrate loading [3]. Patient education targeting emotional stress and preoperative anxiety has been shown to improve patient satisfaction, increase knowledge level, boost patient wellbeing, and benefit surgical outcomes [3,5]. In many cases, the ERAS process can even include primary care providers, who can offer preoperative education and counseling and contribute to the improvement of surgical outcomes [5]. Importantly, ERAS places less emphasis on prolonged fasting and bowel prep than conventional methods [3,5,12]. ERAS guidelines, unlike standard presurgical practices, encourage stopping solid food six hours before surgery and clear fluids two hours prior [5]. Carbohydrate or protein loading is advised in order to improve metabolic status by increasing insulin sensitivity and reducing postoperative inflammation [5,7]. Insulin resistance is widely accepted as one of the most important factors in determining patient outcomes after surgery [8]. Intraoperatively, the goals are similar to those of traditional methods: pain control, anesthesia, and maintenance of normothermia and euvolemia [3,5]. The protocols notably utilize short-acting anesthetic agents that facilitate accelerated recovery, regional analgesic techniques, and minimize reliance on opioids [3,5]. Postoperatively, pain management involves continued use of regional analgesia as well as non-opioid oral analgesics such as nonsteroidal anti-inflammatory drugs (NSAIDs) [3]. The postoperative course also aims to restrict use of nasogastric tubes, initiate early enteral feeding, minimize nausea and vomiting, and encourage early oral nutrition and mobilization [3,9,12]. Success of ERAS is measured by considering outcomes such as length of stay, postoperative narcotic demand, need for postoperative transfer to a hospital, and need for urgent care or readmission within 30 days of surgery [3].

Efficacy of ERAS

ERAS programs have been widely shown to speed up recovery and reduce complications in both open and laparoscopic colorectal surgery [4,9,12,13]. Implementation of the protocols has also been shown to reduce time to regular diet and decrease length of stay by over two days in cases of ventral hernia repair [9]. Similar efficacy has been demonstrated in spine surgery [7,8]. Despite the fact that ERAS has only recently been introduced into the field of neurosurgery, this finding of Dietz et al. is promising considering the anticipated increased volume of these cases in coming years in the setting of an aging United States population [7]. Implementation of ERAS into spine surgery has already demonstrated an associated decrease in not only length of stay but also nursing costs and operative time [7,8].

More recently, ERAS has been applied to acute appendicitis, a common gastrointestinal condition that, even in mild cases, frequently results in hospital admission. Admission for laparoscopic appendectomy (LA) with next-day discharge is currently the standard of care for non-perforated acute appendicitis, but this approach frequently leads to hospital stays ranging in length from 1.8 to 2.88 days. Trejo-Avila et al. recently conducted a prospective, randomized controlled clinical trial of 108 patients that showed that use of an ERAS protocol is associated with reductions in postoperative pain, length of stay, and time to resuming diet. Preoperative care with this ERAS protocol included crystalloid isotonic solution, antibiotics, standard gastric prophylaxis with omeprazole, and opioid-sparing analgesia with ketorolac and acetaminophen. Intraoperatively, patients received general anesthesia with infiltration of all port sites with 0.5% bupivacaine and anti-emesis prophylaxis with dexamethasone and ondansetron. Postoperative pain was managed with ketorolac and acetaminophen while nausea was managed with ondansetron. This approach differed significantly from conventional care, which did not feature infiltration of port sites and employed opioid analgesics such as tramadol for both preoperative and postoperative pain management. ERAS enabled ambulatory management in 90% of patients in this study [4].

The implementation of ERAS has also been tested not only in combination with minimally invasive surgery such as laparoscopic procedures but also with postoperative use of telemedicine. In fact, it has been demonstrated that the trimodal combination of minimally invasive surgery with ERAS and a structured telemedicine program reduced postoperative length of stay, readmission, and emergency department visits. In these scenarios, telemedicine augments the ERAS protocol by allowing close monitoring of patients following discharge and enabling prompt intervention for complications [13].

Non-opioid multimodal therapy

The ideal anesthetic and analgesic techniques for ambulatory surgery should enable both open and endoscopic procedures while allowing rapid postoperative recovery and wide safety margins for the majority of patients [1]. Multimodal analgesia aims to not only optimize pain control but also to manage early gut function in order to decrease insulin resistance and restore homeostasis. One of the key features of ERAS is the use of multimodal analgesia and targeted education of patients regarding pain rating scales used in the postoperative setting as well as alternatives to opioid medications. It has been shown that patients who attended a hospital-based preoperative education class prior to undergoing joint replacement surgery were better able to manage their pain after surgery. This result suggests that especially in the ambulatory setting, expectations for postoperative activity, mobilization, and timing of discharge should be thoroughly discussed [3].

Analgesia

As previously stated, one of the central tenets of ERAS is minimizing, and ultimately eliminating, use of opioids [3,5]. The approach to this goal, especially in ambulatory settings, is through the use of multimodal analgesia with reliance on opioids only for rescue [9]. Studies in obstetrics and gynecology have shown that implementation of ERAS programs for cesarean delivery reduces both inpatient and outpatient opioid use [14]. Multimodal analgesia is a pain management strategy that combines medications with different mechanisms of action and favorable side-effect profiles [3]. In cases where opioids are used, an effort is made to opt for short-acting opioids such as sufentanil [7]. Protocols for multimodal analgesia include the use of agents such as NSAIDs, preoperative gabapentin, intravenous acetaminophen, and ketorolac [3]. For example, combining intraoperative ketamine and ondansetron with postoperative NSAIDs has been shown to decrease opioid consumption, nausea and sedation, and time to mobilization [7]. One of the unique features of ERAS is that its approach to multimodal anesthesia extends beyond the use of medical therapy and also integrates regional anesthesia [3]. The exact protocols for preoperative, intraoperative, and postoperative analgesia follow ERAS guidelines but vary from site to site and are tailored to patient and team preferences [15].

Pharmacotherapy

Acetaminophen is one of the most commonly used medications in ERAS protocols because of its complex mechanism of action and minimal side effects [16]. When used alone, it has been shown to effectively reduce total perioperative opioid requirements in patients undergoing laparoscopic surgery [17]. The effects of acetaminophen are augmented when it is used in combination with NSAIDs [18]. In fact, many ERAS protocols now recommend regular scheduled doses of acetaminophen and NSAIDs [5]. Beyond faster onset and more predictable absorption, no significant difference in efficacy has been consistently observed between the intravenous and oral forms so long as patients are able to tolerate oral dosages [19]. Some ambulatory centers still opt to administer acetaminophen intravenously before surgical incision and transition patients to the oral form after surgery [3].

NSAIDs and cyclooxygenase 2 (COX-2) inhibitors are popular in ambulatory ERAS and work by reducing inflammation. Ibuprofen and ketorolac non-selectively inhibit both COX-1 and COX-2 while celecoxib specifically targets COX-2. Ketorolac in particular has been found especially useful in facilitating a comfortable and accelerated recovery when used in minimally invasive procedures [20]. The combination of ibuprofen (1200 mg/day) and celecoxib (400 mg/day) has been shown to significantly reduce the need for rescue analgesia in the ambulatory surgical setting. In patients undergoing outpatient orthopedic surgery, single-dose celecoxib was shown to produce comparable analgesia to hydrocodone/acetaminophen [3]. Administration of 400 mg celecoxib prior to surgery and for three days postoperatively has been demonstrated to not only control pain but also to hasten recovery following plastic surgery procedures [21].

Gabapentin and pregabalin are gamma-aminobutyric acid analogs with a range of properties including anxiolysis and analgesia. Use of pregabalin 150 mg or 300 mg has been shown to decrease both reported pain and postoperative opioid requirements following laparoscopic surgery. Combined administration of gabapentin with acetaminophen is efficacious at decreasing pain following spine surgery [22]. Gabapentin and pregabalin are both generally well tolerated, but side effects of sedation and dizziness should be monitored more closely in higher-risk patients such as the elderly [23].

Although most ERAS sites do not recommend use of sedatives prior to surgery, one of the approaches to multimodal analgesia involves the use of sedatives to complement local analgesia [1,5]. Some departments have combined preoperative agents like midazolam with local anesthesia to achieve “sedoanalgesia”. Midazolam also offers the benefit of serving as an anxiolytic, which is highly desirable for many patients undergoing surgery. Unfortunately, midazolam still has the potential to sub-optimally prolong postoperative recovery. To address this problem, flumazenil, an agent the reverses the sedative effects of benzodiazepines, has been shown to be effective at a postoperative dose of 0.5 intravenously in reversing sedation due to midazolam and enabling patients to be ready for discharge within 15 minutes of surgery. Use of flumazenil has not been shown to significantly increase perception of postsurgical pain. The tissue distribution of flumazenil also decreases risk of re-sedation with single-dose administration despite its short half-life [1].

Systemic intravenous lidocaine has anti-inflammatory and analgesic properties, is easy to use, and has minimal toxicity at typical doses (i.e., 2 mg/kg/h intraoperatively continued at 1.33 mg/kg/h for 24 hours postoperatively). Although it is less commonly used in ambulatory settings, systemic lidocaine infusions have been shown to decrease postoperative opioid consumption in patients following laparoscopic colectomy [24]. When used for inpatient ERAS, intravenous lidocaine decreases length of stay and postoperative nausea and vomiting (PONV) [25]. Given its lower popularity as an ERAS agent compared to other pharmacotherapy options, further study of patient outcomes is needed to make firm recommendations [3].

Regional Anesthesia

The American Society of Anesthesiologists Task Force on Acute Pain Management endorses the integration of anesthesia-based techniques into ERAS protocols [3]. While at one point epidural analgesia was thought to be a useful alternative for limiting opiate use following laparotomy incisions, this approach was shown to contribute to hypotension and PONV, delayed removal of urinary catheters, and limited mobility during the postoperative period [5,7]. Ultrasound guidance has made techniques like regional nerve blocks, neuraxial anesthesia, and wound infiltration easier to perform in everyday practice and especially in ambulatory settings [3]. Use of regional anesthesia has been shown to improve several ERAS endpoints, including unwanted effects of opioids such as respiratory and central nervous depression [10].

The transversus abdominis plane (TAP) block has shown promise in minimizing reliance on opioids following inpatient laparoscopic surgery [3,26]. The TAP block provides analgesia to the anterior abdominal wall through infiltration of local anesthesia between the transversus abdominis and internal oblique muscles [3]. The block is commonly performed using liposomal bupivacaine suspension [9]. Infiltration with liposomal bupivacaine in and around surgical sites has been effective in significantly reducing reported pain scores [7]. Liposomal bupivacaine has been shown to provide bettered extended analgesia without healing impairment compared to standard bupivacaine preparations [7]. The TAP block has also been shown to decrease postoperative pain scores and opioid consumption in microvascular breast reconstruction and abdominal wall reconstruction [3]. The analgesic effects of the block are also sometimes supplanted with postoperative scheduled acetaminophen and gabapentin [9]. Although TAP blocks have not been demonstrated to cause significant local anesthetic toxicity and likely pose little risk to the patient even in ambulatory settings, the outpatient use of TAP blocks requires further study before it becomes routinely implemented [3].

The paravertebral block (PVB) is a type of chest wall block that is considered the gold standard for regional anesthesia for breast surgery [3,26]. Use of PVB in conjunction with general anesthesia has been demonstrated to result in decreased pain ratings, decreased postoperative opioid consumption, and overall improved recovery compared to use of general anesthesia alone. The major risk of PVB is pneumothorax, but rates are exceedingly low especially with widespread use of ultrasound guidance [3]. Fascial blocks such as the pectoralis, serratus, and erector spinae blocks represent new alternatives to PVB that have been shown to reduce opioid consumption following breast surgery [3,26,27]. To date, studies supporting their use in same-day surgery have been small and more data is needed to support their quality and safety compared to PVB [3].

Antiemetics

PONV has been shown to increase time to mobilization, resumption of diet, and return to normal function [5]. Thus, preventing and efficiently treating PONV is an important component of ERAS [3,5]. PONV presents significant concerns for patients undergoing ambulatory surgery given lack of access to effective antiemetic therapy following discharge [3]. As with analgesia, a multimodal approach is recommended for treatment [5]. Given that short-acting antiemetics have not been shown to be effective, administration of a standardized antiemetic protocol consisting of dexamethasone and a serotonin receptor antagonist like ondansetron is favored for the majority of patients. This protocol can also be augmented by the addition of aprepitant, a neurokinin-1 (NK-1 ) receptor antagonist, for high-risk patients [3]. It has been proposed that in the future, genetic techniques can be used to identify patients with genetic predispositions to PONV in order to preemptively provide them with augmented, scheduled therapy [3,5].

Limitations of ERAS

As mentioned, while the expansion of ERAS guidelines from elective surgery to emergency surgery is being explored, certain limitations are difficult to overcome [4]. For instance, one of the major ERAS recommendations for preoperative optimization is to increase exercise and decrease smoking and alcohol intake - recreating this optimization exactly in acute care settings is impossible [4,5].

It is important to note that presently, there exist limitations even to implementing ERAS for certain elective procedures. Large-scale analysis of 85,321 cases of laparoscopic sleeve gastrectomy (LSG) from the Metabolic and Bariatric Surgery Accreditation and Quality Improvement Program database by Inaba et al. demonstrated that attempts at same-day discharge are associated with increased complications, readmissions, and reoperations compared to discharge on postoperative day one [2]. Similarly, Colvin et al. found that benefits of ERAS are limited in patients undergoing complex hernia repairs as their study found no change in duration of stay [9]. It is thought that monitoring patients overnight not only allows providers to detect and provide early intervention for postoperative issues such as respiratory deconditioning, but also allows ample time for patient education on best practices after surgery [2]. Although length of stay has been an important measure in research of inpatient ERAS, some believe that ERAS in ambulatory settings should focus less on ERAS and more on pain management, early mobilization, and overall quality of recovery [3].

One of the other difficulties associated with ERAS is reliably predicting which patients could safely undergo surgery in an ambulatory setting [4,28]. The inability to accurately differentiate between low-risk and high-risk patients has been frequently cited as a factor that contributes to poor outcomes at ambulatory centers and with same-day discharge [2]. In looking at patients who could qualify for ambulatory appendectomy, the Saint-Antoine Score, a predictive score of early postoperative discharge, was created. This score considered BMI, total leukocyte count, C-reactive protein, lack of radiological evidence of perforation, and appendix diameter less than 10 mm. Use of this system had a success rate of 97%, suggesting that predictive scoring may prove useful in transitioning ERAS to more settings [28]. A similar disease-specific scoring system, the HerQIes score, is used to evaluate quality of life in patients with ventral hernias prior to surgery and assess patient risk [9]. Other scoring systems have aimed to quantify not only patient risk but also patient qualities that may slow the course of recovery [29]. Such predictive scoring systems could help identify patients with heart disease or obstructive sleep apnea [28]. In cases where such patients are identified, especially those with elevated hemoglobin A1c (HbA1c), exclusion is not necessarily the only option since surgery can be delayed with concomitant referral to specialists such as endocrinologists [7].

## Conclusions

Implementation of optimized ERAS protocols consistently demonstrates successfully efficiency and outcomes across several fields including orthopedics, general surgery, neurosurgery, urology, and plastic surgery. One of the keys to ERAS and especially decreasing postoperative opioid consumption is the use of appropriate patient selection criteria and sufficient patient counseling and education. Multimodal analgesia combined with regional anesthesia are effective in delivering opioid-sparing pain management. When used alongside prophylactic treatment for postoperative nausea and vomiting, these techniques significantly improve patient satisfaction rates. Since the basic aim of ERAS is to decrease the stress of surgery on the body, the goal should be to apply these guidelines to as many eligible patients undergoing surgery as possible.
